# Application of the indirect fluorescent antibody assay in the study of malaria infection in the Yangtze River Three Gorges Reservoir, China

**DOI:** 10.1186/1475-2875-8-199

**Published:** 2009-08-13

**Authors:** Wang Duo-Quan, Tang Lin-Hua, Gu Zhen-Cheng, Zheng Xiang, Yang Man-Ni

**Affiliations:** 1 National Institute of Parasitic Disease, Chinese Center for Disease Control and Prevention, WHO Collaborating Center for Malaria, Schistosomiasis and Filariasis, Shanghai 200025, PR China

## Abstract

**Background:**

China Yangtze Three Gorges Project (TGP) is one of the biggest construction projects in the world. The areas around the Three Gorge Dam has a history of tertian malaria and subtertian malaria epidemic, but there are no overall data about malaria epidemics before the completion of the project. The objective of this study was to get a reliable baseline on malaria infection in the Yangtze River Three Gorges reservoir area and to provide reference data for future studies about the impact of the project on malaria epidemics.

**Methods:**

Two surveys of malaria infection were carried out in area, at six-month intervals in May and October 2008. About 3,600 dual specimens blood film samples for parasite diagnosis and filter paper blood spots for serology (using the immunofluorescence antibody test) were collected from the general population, including school populations, whenever possible.

**Results:**

The overall percentage of positive response of the same population during post-transmission periods was about twice (1.40/0.72) of that in pre-transmission. Positive individuals under 15 years of age were detected in all the localities.

**Conclusion:**

A certain extent of malaria infection existed in this area. Additional studies are needed to determine the length of malaria experience, and chemotherapeutic intervention as well as the distribution of main vectors for transmission in this area.

## Background

China Yangtze Three Gorges Project (TGP), as one of the biggest hydropower-complex projects in the world, is located at latitude 29° ~ 31° 50 ', longitude 106° 20' ~ 110° 30 ', including 25 county-level divisions of Chongqing municipality and Hubei province and with the total population of 16 million. The mountainous areas represent 74% of the region only with 4.3% plain area in the river valley and 21.7% hilly area. The climate of the reservoir region of the Three Gorges Project is the subtropical monsoon climate. Three Gorges Project water level reached 172.3 meters above sea level elevation in 2008 and the project will be completed in 2009[1]. Although improved hydraulic infrastructure holds potential for alleviating poverty, promoting economic growth, improving food security and mitigating floods, adverse health effects may undermine these objectives [2]. Indeed, dams in Cameroon [3], Kenya [4] and Mali [5] have resulted in an increased malaria burden, a trend that appears to hold for small Ethiopian dams [6]. The area around the Three Gorge Dam has a history of tertian malaria and subtertian malaria epidemic. There was no subtertian malaria after 1960, and the prevalence was controlled by the end of 1980s. The principal transmitting vectors were *Anopheles sinensis *and *Anopheles anthropophagus*, while the transmission vector was only *An. sinensis *[7]. No systematic, overall, data about malaria epidemics was specified before the overall completion of the project.

The immunofluorescent antibody test (IFA) is a reliable and reproducible procedure for determining antibody levels for the indication of current or past infection with malaria [8]. All the reported infections in the region were due to *Plasmodium vivax *and about 80% infections were symptomomatic, while the rest was detected by microscopy diagnosis: this was less than 1/10,000 in recent years, but the traditional surveillance through passive case detection provides information which may be inadequate. The addition of a serology method, such as IFA, may be of considerable value in understanding the actual epidemic situation in this area. Therefore, the study was designed to provide a reliable assessment of malaria infection in the area as well as the baseline data for further study about the influence on malaria epidemic in the future from the great project.

## Methods

### Study area and subjects

**Figure 1:**
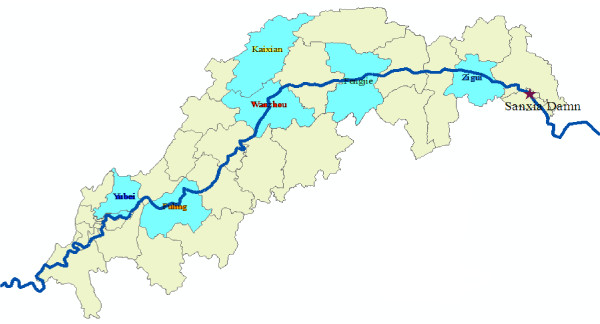
**Location of study areas (blue color) in Three Gorges Reservoir Area**.

According to the diversity of environment as well as its recent malaria incidence, a stratified random unequal proportion sampling method was used in **t**his study to provide a representative sample of the civilian population in the Three Gorges Reservoir area of the Yangtze River. Six counties (including 24 villages) and more than 3,600 subjects were selected from the upper reaches and mid-stream as well as downstream of the Three Gorges reservoir area (Figure 1). Each village was of at least 150 persons, including school populations whenever possible, because most of the school populations were under 15 years of age [9], assuming that such a study would reflect the local infection. Two surveys of collecting filter paper blood spots for determination of IFA response were conducted in the same local population, at a six-month interval during the period of May to October 2008.

### Sampling collecting

The samples consisted of a thick blood film (only in the second survey) for parasite diagnosis and a dual filter paper sample for serological diagnosis, both derived from a single finger prick collection. More than 3,600 such dual samples were thus collected. The filter paper samples were obtained by filling two delineated circular (about 0.5 inch in radius) areas on a filter paper strip and its subsequent volume/dilution was 1:20. The filter paper samples were prepared by expressing drops of blood from finger puncture directly onto the paper, with enough volume to completely fill the delineated circular area. This method has been shown to collect approximately double the amount of blood than the haematocrit tube method, and the implication relative to the IFA titers will be discussed below.

### Sampling detection

Thick films from the selected subjects were made on each slide and the blood films thoroughly dried and stained by the standard Giemsa method, usually within 48 hours, but less than 96 hours after they were taken. Examinations were done in National Institute of Parasitic Disease, Chinese Center for Disease Control and Prevention, WHO Collaborating Center for Malaria, Schistosomiasis and Filariasis by a senior technician. Examination of more than 200 oil immersion fields was required to verify negativity of each blood film.

Filter papers were thoroughly dried and placed in plastic bags, and were kept refrigerated to the extent possible prior to their return to the lab in Shanghai. The paired samples were cut apart, coded and placed in a freezer at -20°C until transported to the CDC laboratory by EMS for IFA processing. The IFA test was done according to the standard methods of Collins and co-workers [10], using antigens of *Plasmodium cynomolgy *to test the level of IgG in the serum.

Subsequent to the first survey identical normal and malaria-infected control sera were included as part of each test run throughout the study in order to verify the specificity and reproducibility of the test. Titers of 1:20 or higher were considered to be positive and evidence of previous malaria experience. Most studies in China, such as [9,11] substantiated that the increased titers could indicate whether the subject had been infected.

### Statistical method

Database was set up and analysis by SPSS16.0 package. The prevalence was used and comparison was used by χ^2 ^test and the study was approved by the Ethical Committee of the Ministry of Health.

## Results

Table 1 showed that the reported incidence of malaria from Case Reporting Information System was below 1/100,000 during 2004–2007 in the selected county. The result indicated the low incidence of malaria in this area.

**Table 1:** The reported incidence of malaria from Case Reporting Information System between 2004 to 2007 in Three Gorges Reservoir Area (1/100,000)

Locality	2004 Year		2005 Year		2006 Year		2007 Year	
	
	No.cases	Incidence	No.cases	Incidence	No.cases	Incidence	No.cases	Incidence
Fengjie	0	0	2	0.25	0	0	1	0.13
Kaixian	6	0.45	1	0.08	2	0.15	1	0.08
Wanzhou	0	0	3	0.19	1	0.06	4	0.25
Fuling	1	0.09	2	0.18	2	0.18	0	0
Yubei	2	0.26	2	0.26	0	0	1	0.13
Zigui	0	0	1	0.26	0	0	0	0

Total	9	0.15	11	0.19	5	0.08	7	0.12

**Figure 2:**
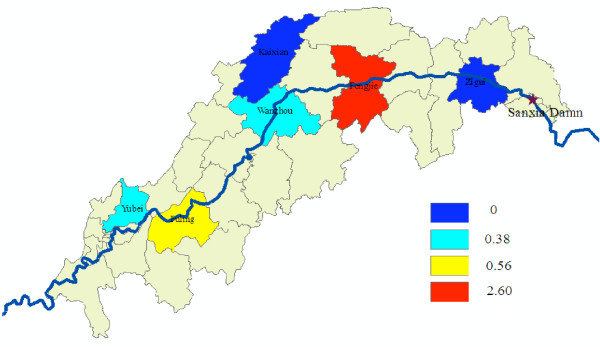
**The percentage of positive results under 15 years of age in the study areas in May**.

The summary of the serologic results of the surveys is presented in Table 2. Samples were collected from 3,613 individuals during visits to all houses in the villages and schools. Twenty-six positive cases were found, in which more than 90% of all positive titers (1:20) occurred in the 15-and-over age group. The percentage of positive results in the 15-and-over age group from different localities was similar; while there appeared to be wide difference (Figure 2) under 15 years of age in the region; no positive ones were seen in Kaixian as well as Zigui, but Fengjie gave the highest percentage of positive, which maybe related to the fewer samples detected.

**Table 2:** Serology (IFA) result with *Plasmodium cynomolgi;* Three Gorges Reservoir Area of the Yangtze River, China, May 2008

Locality	Age group	No. sera	Positive	
			
			N	%
Fengjie	<15	39	1	2.6
	≥15	557	4	0.72
Fuling	<15	179	1	0.56
	≥15	439	4	0.91
Kaixian	<15	111	0	0
	≥15	489	4	0.83
Wanzhou	<15	260	1	0.38
	≥15	340	0	0.00
Yubei	<15	264	1	0.38
	≥15	336	1	0.30
Zigui	<15	207	0	0
	≥15	392	9	2.30

Total		3613	26	**0.72**

**Figure 3:**
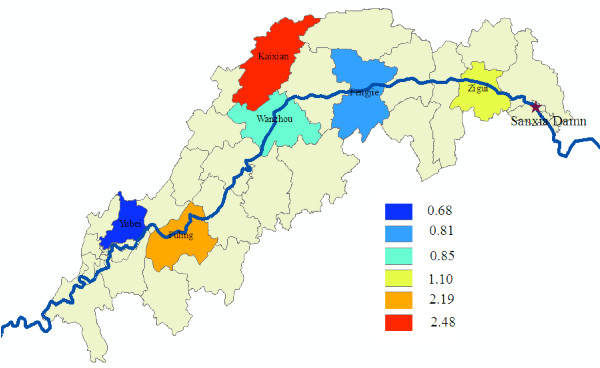
**The percentage of positive results under 15 years of age in the study areas in October**.

As indicated in Table 3, 3,800 samples were collected and examined for the detection of malaria parasites and antibody in October 2008. The overall percentage of positive response in the 15-and-over age group (1.21%) was approximately the same as that of under 15 years of age (1.51%). Positive responses were not evenly distributed among different localities, in which Fuling has the highest percentage of positive while Yubei gave the lowest number (Figure 3).

**Table 3:** Parasitologyandserology (IFA) results using *Plasmodium cynomolgi; *Three Gorges Reservoir Area of the Yangtze River, China, October 2008

Locality	Age group	No. sera	Positive N (%)			
			
			Serology (IFA)		Parasitology	
Fengjie	<15	248	2	0.81	0	0
	≥15	383	3	0.78	0	0
Fuling	<15	228	5	2.19	0	0
	≥15	396	10	2.53	0	0
Kaixian	<15	121	3	2.48	0	0
	≥15	484	7	1.45	0	0
Wanzhou	<15	235	2	0.85	0	0
	≥15	373	12	3.22	0	0
Yubei	<15	294	2	0.68	0	0
	≥15	306	1	0.33	0	0
Zigui	<15	362	4	1.1	0	0
	≥15	370	2	0.54	0	0

Total		3,800	53	1.40	0	0

There was a marked increase in the number of seropositive samples of the total same population during the post-transmission period: the overall percentage of positive response of the same population during post-transmission period was about twice (1.40/0.72) that in pre-transmission, indicating a certain extent prevalence in the region in 2008. An age-related increase in antibody prevalence was evident under 15 years of age and about threefold increase in antibody positive individuals, which the percentage of positive was significant statistically higher (*P *< 0.01) than that of pre-transmission periods. Positive individuals less than 15 years old were detected in all the localities. But no demonstrable parasitaemia was found in this study.

## Discussion

Serological methods can provide additional evidence of the extent as well as degree of malaria endemicity and reflect the period of the infection [12]. Serological techniques have provided valuable epidemiological information, especially in areas with low endemicity [13]. Rates of parasitaemia is the classical method for measuring the endemicity of malaria, while the incidence of parasitaemia alone can completely fail to provide an adequate picture of the pattern of malaria in a population. When the incidence of malaria is low, mass blood surveys do not yield results commensurate with the work involved [10]. Domestic study [14] has also confirmed that when the incidence of malaria was about 2%, the parasitaemia rate surveys should not reflect the change of prevalence, while the application of fluorescent antibody could reflect the situation. The results of this study also shown that, although none had been detected parasite positive in the population tested, the population had a certain antibody level with significant changes in 2008. The result that a marked increase in the number of sero-positive samples of the total same population during post-transmission periods supported the certain extent infection of malaria in 2008. It was assumed that evidence of active transmission of malaria in the population would be indicated by the presence of positive IFA titers in the younger segments of the population (<15 years of age) [15]. In this study, positive individuals under 15 years of age were detected in all the localities and the positive percentage in October was significant (*P *< 0.01) higher than that in May during 2008, reflecting no interruption of malaria transmission in this area. While one can argue that the IFA test may not be very specific, previous studies have revealed an acceptable specificity [16,17]. The second possible explanation may be that there is simian malaria, which could cause the cross-positivity, further study should be carried out for surveying the simian malaria though no case reported before in this area.

Although the reported incidence of malaria from Case Reporting Information System was below 1/100,000 during 2004–2008, a certain extent malaria infection level was confirmed in the area. The reason that the incidence from Case Reporting Information System was far below the infection rate of the study may be as follows: (1) with the degree of infection decline with the lower incidence of the population, few such infection would be expected to produce clinical symptoms causing a visit to the voluntary collaborator; (2) because of dwindling resources and support for malaria eradication programme, many villages are having to reorient priorities and to determine the needs for continuing malaria control measures in low incidence areas which lead to the reduction of malaria prevention and control capacity in the rural area, therefore, the information from traditional surveillance through passive case detecting may be inadequate; (3) the average rate of right answer about malaria basic knowledge was about 3.2%(116/3613) of the total surveyed population. Above all, although the trend of malaria endemic in the region has been effectively controlled in recent years, research reports as well as surveillance data of recent 40 years confirmed the history of the region was a variety of natural foci of disease and insect-borne infectious disease endemic area [18]. The result from the surveillance spot showed that *An. sinensis *was the main vector for transmission of malaria in the local area. With the Three Gorges Dam reservoir water level increase and slowing down water flow as well as the expansion of environmental water area, the ecological distribution of malaria transmission vector may change; the importation of the source of infection will increase with development of the local tourism because of the Great Dam, so it is essential that malaria surveillance be continued and strengthened in the area because of extensive vector distribution, water slowdown, surface widened and eliminate stagnant water-belt formation which may provide favourable conditions for the breeding of anopheles after the Three Gorge Dam construction.

## Conclusion

The IFA test is applicable for the determination of antibody levels to malaria. A certain extent of malaria infection was confirmed in the Three Gorges Reservoir area of the Yangtze River by this study, regardless of the low reported incidence. With the ecological change following Three Gorges Dam building, a strengthening of malaria surveillance is urgent. Above all, the study provided a reliable indication of malaria infection in the area, which will be the reference data for further study about the influence on malaria epidemic from the project. However, additional studies are needed to determine the length of malaria experience, and chemotherapeutic intervention as well as the distribution of main vector for transmission of malaria in this area. With this information, the level of transmission in the area could be more accurately ascertained.

## Conflict of interests

The authors hereby certify that no conflict of interest of any kind occurred in the framework of this study.

## Authors' contributions

TLH and WDQ organized the survey, coordinated and supervised the field work and the data entry. GZC helped in the study design and choice of study villages to be included in the trial and reviewed the manuscript. ZX and YMN processed all the filter papers with the IFAT and performed the quality controls of all blood samples results.
